# BRAFV600E Negatively Regulates the AKT Pathway in Melanoma Cell Lines

**DOI:** 10.1371/journal.pone.0042598

**Published:** 2012-08-03

**Authors:** Brenden Chen, Christine Tardell, Brian Higgins, Kathryn Packman, John F. Boylan, Huifeng Niu

**Affiliations:** Discovery Oncology, Hoffmann-La Roche Inc., Nutley, New Jersey, United States of America; Children's Hospital Boston & Harvard Medical School, United States of America

## Abstract

Cross-feedback activation of MAPK and AKT pathways is implicated as a resistance mechanism for cancer therapeutic agents targeting either RAF/MEK or PI3K/AKT/mTOR. It is thus important to have a better understanding of the molecular resistance mechanisms to improve patient survival benefit from these agents. Here we show that BRAFV600E is a negative regulator of the AKT pathway. Expression of BRAFV600E in NIH3T3 cells significantly suppresses MEK inhibitor (RG7167) or mTORC1 inhibitor (rapamycin) induced AKT phosphorylation (pAKT) and downstream signal activation. Treatment-induced pAKT elevation is found in BRAF wild type melanoma cells but not in a subset of melanoma cell lines harboring BRAFV600E. Knock-down of BRAFV600E in these melanoma cells elevates basal pAKT and downstream signals, whereas knock-down of CRAF, MEK1/2 or ERK1/2 or treatment with a BRAF inhibitor have no impact on pAKT. Mechanistically, we show that BRAFV600E interacts with rictor complex (mTORC2) and regulates pAKT through mTORC2. BRAFV600E is identified in mTORC2 after immunoprecipitation of rictor. Knock-down of rictor abrogates BRAFV600E depletion induced pAKT. Knock-down of BRAFV600E enhances cellular enzyme activity of mTORC2. Aberrant activation of AKT pathway by PTEN loss appears to override the negative impact of BRAFV600E on pAKT. Taken together, our findings suggest that in a subset of BRAFV600E melanoma cells, BRAFV600E negatively regulates AKT pathway in a rictor-dependent, MEK/ERK and BRAF kinase-independent manner. Our study reveals a novel molecular mechanism underlying the regulation of feedback loops between the MAPK and AKT pathways.

## Introduction

The MAPK and AKT pathway represent the most frequently mutated signaling pathways in human cancers. The high prevalence of dysregulation of these two pathways has provided a rationale for the development of target-based therapeutics for cancer treatment. In malignant melanoma, more than 50% of tumors carry BRAFV600E mutation and 70% have elevated AKT phosphorylation and/or activated mTOR activities [1]–[3].

BRAF inhibitor vemurafenib has shown remarkable clinical efficacy for the treatment of metastatic or unresectable melanoma with a BRAF V600E mutation [4]. Various MEK inhibitors and PI3K/AKT/mTOR inhibitors are currently in clinical development, either as monotherapy or in combination therapy, for the treatment of various cancers [5]–[9]. However, patient survival benefits are likely limited due to a rapid acquisition of drug resistance [10]–[16]. Rapamycin (mTORC1 inhibitor) abrogates intrinsic negative feedback of AKT/mTOR and MEK/ERK and induces AKT and MEK/ERK phosphorylation [17], [18]. Similarly, MEK inhibitors abolish the same negative feedback loops, leading to induction of MEK and AKT phosphorylation [19], [20]. Feedback induction of MEK and AKT phosphorylation has been thought to confer resistance and limit the clinical activity of these agents. To design improved therapeutic strategies, a more thorough understanding of the complex internal feedback loops and crosstalk between the two pathways is required.

In this study, we identified a novel crosstalk mechanism between the two pathways, in which BRAFV600E negatively regulates AKT pathway. This mechanism provides a potential explanation why a limited subset of BRAFV600E melanoma cells are exquisitely sensitive to MEK inhibition and supports the rationale combination of AKT and MEK inhibition as a viable cancer therapeutic strategy.

## Results

### MEK inhibitor induces AKT phosphorylation in NIH3T3 cells but not in NIH3T3 expressing BRAFV600E

**Figure 1:**
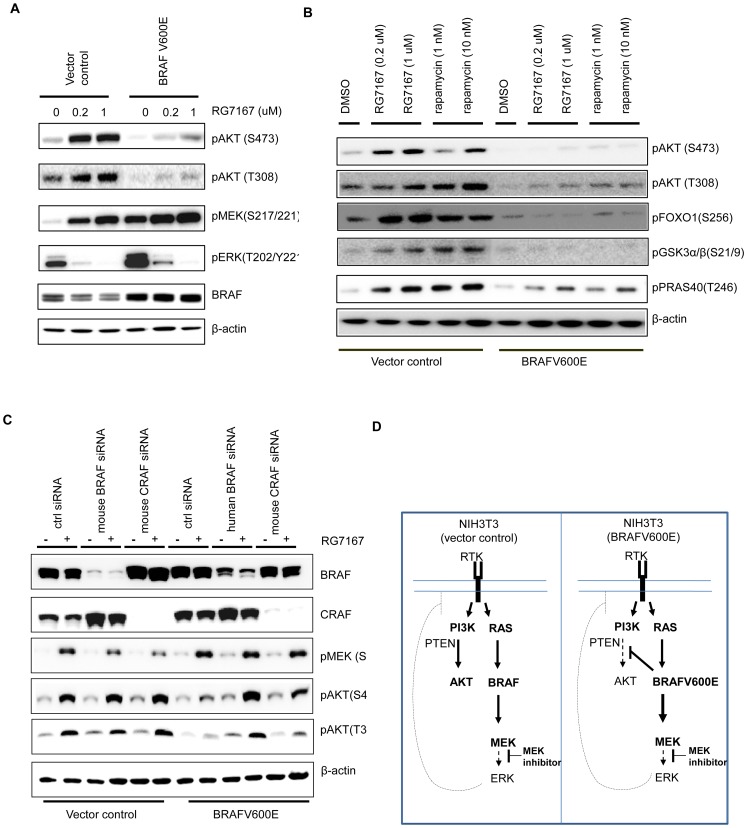
Treatment with MEK inhibitor RG7167 or rapamycin activates AKT pathway in NIH3T3 vector control clones but not in BRAFV600E clones. (A) Western blot analysis of AKT, MEK, and ERK phosphorylation in the isogenic pair of NIH3T3 clones 4 hours post treatment with MEK inhibitor RG7167 at indicated concentration. (B) Western blot analysis of AKT substrates (FOXO1, GSK3α/β, PRAS40) phosphorylation in the isogenic pair of NIH3T3 cells 4 hours post treatment with either MEK inhibitor RG7167 or rapamycin at indicated concentrations. (C) Western blot analysis of MEK and AKT phosphorylation in the isogenic pair of NIH3T3 cells 24 hours after CRAF or BRAF knock-down and 4 hours post RG7167 treatment. (D) Schematic presentation of a model of the cross-talk between MAPK and AKT pathways in engineered NIH3T3 clones. The left panel - MEK inhibitor induces pAKT by suppressing ERK-dependent negative feedback loop in control NIH3T3 cells. The right panel – although MEK inhibitor should induce pAKT through alleviating ERK-dependent feedback loop, the presence of BRAFV600E imparts a negative impact on pAKT induction.

Several cross-feedback loops are reported to regulate MAPK and AKT pathways [17]–[22]. Consistent with these studies, treatment with the MEK inhibitor RG7167 (RO4987655) [23] or the mTORC1 inhibitor rapamycin in NIH3T3 cells strongly induced pAKT, at both Ser473 and Thr308 (Fig. 1A). The induction of pAKT by RG7167 could be seen within 1 hour of treatment (Fig. S1). Moreover, the pAKT induction subsequently led to phosphorylation of AKT substrates, indicating an activation of AKT pathway (Fig. 1B). When human BRAFV600E was stably expressed in NIH3T3 cells, BRAFV600E activated MEK/ERK phosphorylation and stimulated cell growth both *in vitro* and *in vivo* (Fig. S2). In these cells, induction of pAKT by either compound was significantly reduced (Fig. 1A, Fig. S1). This reduction in pAKT elevation was not due to insufficient suppression of MAPK pathway signaling, as in both cells, ERK phosphorylation was significantly suppressed. The reduction of pAKT elevation in NIH3T3 (BRAFV600E) cells also translated into a loss of AKT substrates phosphorylation (Fig. 1B), suggestive of a suppressed AKT pathway activity in the presence of BRAFV600E. To further demonstrate the role of BRAFV600E in regulating pAKT, we knocked down BRAF (wild type or V600E) in these cells using siRNA. Compared to control cells where knock-down of endogenous wild type BRAF showed no effect on MEK inhibitor-mediated induction of pAKT, in NIH3T3 (BRAFV600E) cells, knock-down of exogenous human BRAFV600E restored the MEK inhibitor-mediated induction of pAKT (Fig. 1C). Knock-down of CRAF, a member of RAF family known to influence BRAF functions through heterodimerization, in both cells had a minimal impact on pAKT, suggesting that CRAF played no major role in the regulation of MEK inhibitor-mediated pAKT induction. Together these observations suggest that BRAFV600E imparts a negative effect on the cross-feedback between the MAPK and AKT pathways (Fig. 1D).

### MEK inhibitor enhances AKT phosphorylation in melanoma cells harboring wild-type BRAF but not BRAFV600E

**Figure 2:**
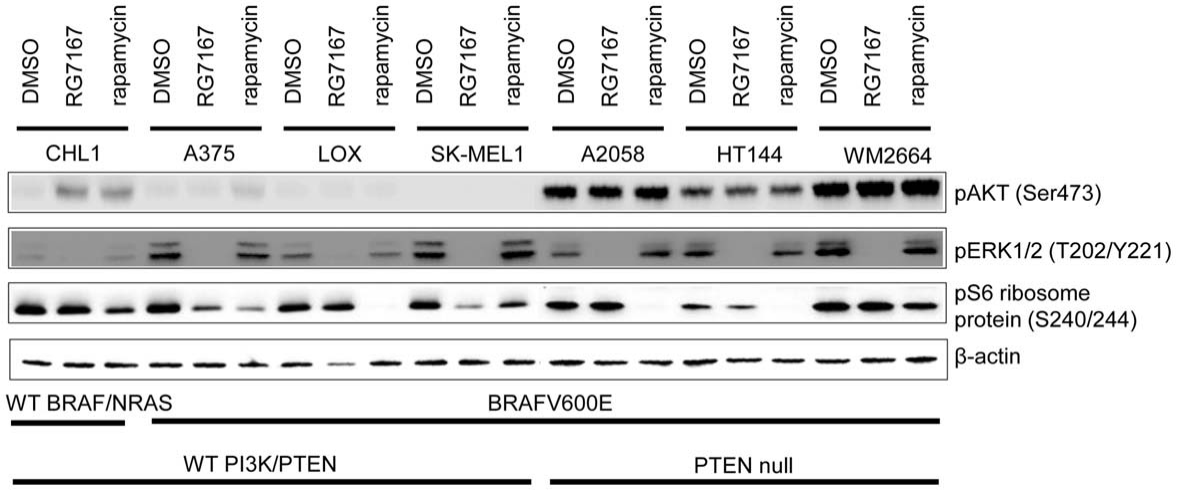
Melanoma cell lines with different genetic backgrounds respond differently to treatment-induced AKT phosphorylation. Western blot analysis of the phosphorylation of AKT, ERK and S6 ribosome proteins in melanoma cell lines 4 hours after treatment with MEK inhibitor RG7167 or rapamycin.

We further tested the effect of BRAFV600E on pAKT in a panel of human melanoma cell lines with different genetic backgrounds. In CHL1 melanoma cell line with wild-type BRAF, MEK inhibitor suppressed ERK phosphorylation and induced pAKT (Fig. 2A). Consistent with published results, rapamycin induced AKT phosphorylation via abrogation of an internal negative feedback loop of AKT pathway and suppressed S6 ribosome protein phosphorylation at Ser240 and 244, which are regulated exclusively by mammalian target of rapamycin complex 1 (mTORC1). [18]. In contrast, in a subset of melanoma cell lines, A375, LOX and SK-MEL1, which harbor BRAFV600E with no concurrent mutations in PI3K or PTEN, MEK inhibitor failed to induce pAKT (Fig. 2A), despite suppression of ERK phosphorylation. Notably, the MEK inhibitor also suppressed S6 ribosome protein phosphorylation at Ser240 and 244, suggesting mTORC1 may be regulated by BRAFV600E in this context. Treatment with rapamycin in these cells did not induce pAKT, suggesting the AKT pathway internal feedback loop may be functionally impaired in the presence of BRAFV600E. Consistent with our observation in NIH3T3 cells, BRAFV600E seems to be able to impart a negative effect on AKT pathway activation in melanoma cells.

As more than 40% of melanoma in clinical samples show a combined elevation of MAPK and AKT signaling, we also tested our hypothesis in a group of melanoma cell lines harboring BRAFV600E and mutated PTEN, which activates AKT pathway. In A2058, HT144 and WM2664, loss of PTEN function led to an elevation of basal AKT phosphorylation. In these cell lines, treatment with either MEK inhibitor or rapamycin did not further enhance pAKT, even though phosphorylation of ERK and S6 ribosome protein were respectively suppressed, suggesting that PTEN mutation not only dominates MEK inhibitor or rapamycin induced feedback activation, but also overrides the BRAFV600E-mediated negative impact on AKT signaling (Fig. 2A). Interestingly, in this subset of melanoma cell lines, treatment with MEK inhibitor did not result in a down-regulation of S6 ribosome protein phosphorylation, suggesting MEK-ERK signaling may not regulate mTORC1 functions in this context, possibly due to a PTEN dominant regulation on the AKT pathway.

**Figure 3:**
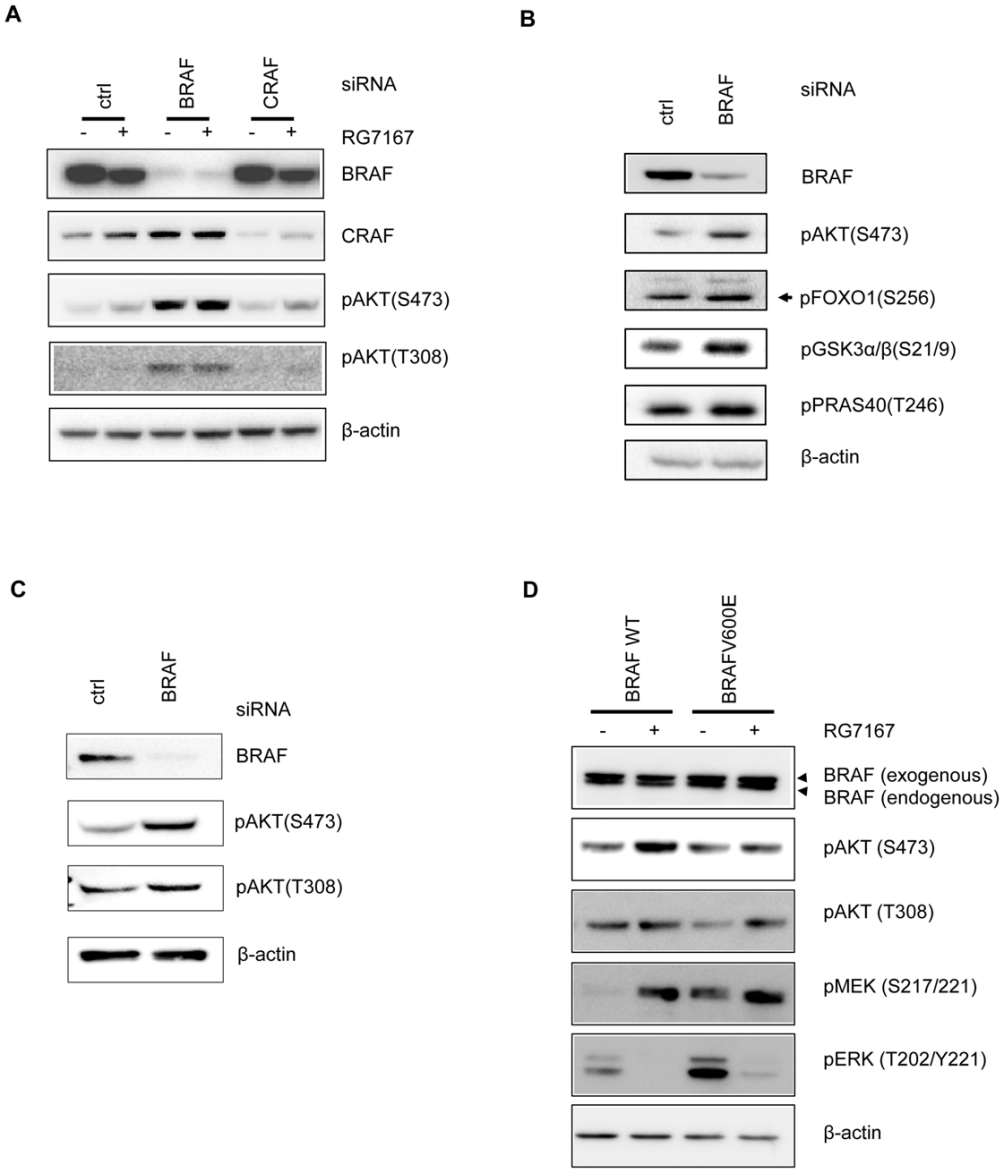
BRAFV600E is required and sufficient to suppress AKT phosphorylation in a subset of melanoma cells. (A) Western blot analysis of AKT phosphorylation 24 hours after BRAF or CRAF knock-down in A375 melanoma cell line 4 hour post RG7167 treatment. (B) Western blot analysis of AKT substrates (FOXO1, GSK3α/β, PRAS40) phosphorylation 24 hours after BRAF knock-down in A375 melanoma cell line. (C) Western blot analysis of AKT phosphorylation 24 hours after BRAF knock-down in LOX melanoma cell line. (D) Western blot analysis of AKT, MEK, and ERK phosphorylation in CHL1 melanoma cell line 24 hours after transient transfection (wild-type BRAF or BRAFV600E) and treated with RG7167 for 4 hours.

To prove that BRAFV600E was indeed required for the lack of induction of pAKT in a subset of melanoma cells, we knocked down BRAFV600E or CRAF in A375 melanoma cells and evaluated the AKT and its substrate phosphorylation. The result showed that knock-down of BRAFV600E significantly elevated basal pAKT at both Ser473 and Thr308. Treatment with MEK inhibitor in BRAF knock-down A375 cells slightly further enhanced pAKT (Fig. 3A). In contrast, knock-down of CRAF had minimal impacts on pAKT. Treatment with MEK inhibitor also failed to further induce pAKT (Fig. 3A). The elevation of basal pAKT by knock-down of BRAFV600E subsequently led to induction of AKT substrates phosphorylation (Fig. 3B). Similar to A375 cells, knock-down of BRAFV600E in LOX cells resulted in an elevation of pAKT (Fig. 3C). In contrast, knock-down of BRAFV600E in A2058 and HT144 did not elevate pAKT, consistent with the idea that PTEN dysfunction dominantly drives the AKT signaling (Fig. S3).

To demonstrate that BRAFV600E is also sufficient to suppress AKT pathway activation in melanoma cells, we transiently expressed BRAFV600E or wild-type BRAF in CHL1 melanoma cells that contain endogenous wild-type BRAF and assessed the induction of pAKT in response to MEK inhibitor treatment. Expression of BRAFV600E, but not wild type BRAF, activated pMEK and pERK in CHL1 cells. MEK inhibitor RG7167 inhibited pERK and induced feedback elevation of pMEK in CHL1 cells expressing either exogenous wild type or mutant BRAF. Treatment with RG7167 induced pAKT in CHL1 cells expressing exogenous wild-type BRAF. In contrast, RG7167-induced pAKT was suppressed in CHL1 cells expressing exogenous BRAFV600E (Fig. 3D). Notably, RG7167 induced pAKT (T308) is less affected by BRAFV600E compared to pAKT (S473) although basal levels of pAKT (T308) were reduced. Taken together, these results suggest that BRAFV600E is able to negatively regulate basal and the MEK inhibitor induced AKT signaling in a subset of melanoma cells without dysregulation of AKT pathway.

### BRAFV600E negatively regulates AKT phosphorylation independent of downstream MEK and ERK and its kinase activity

**Figure 4:**
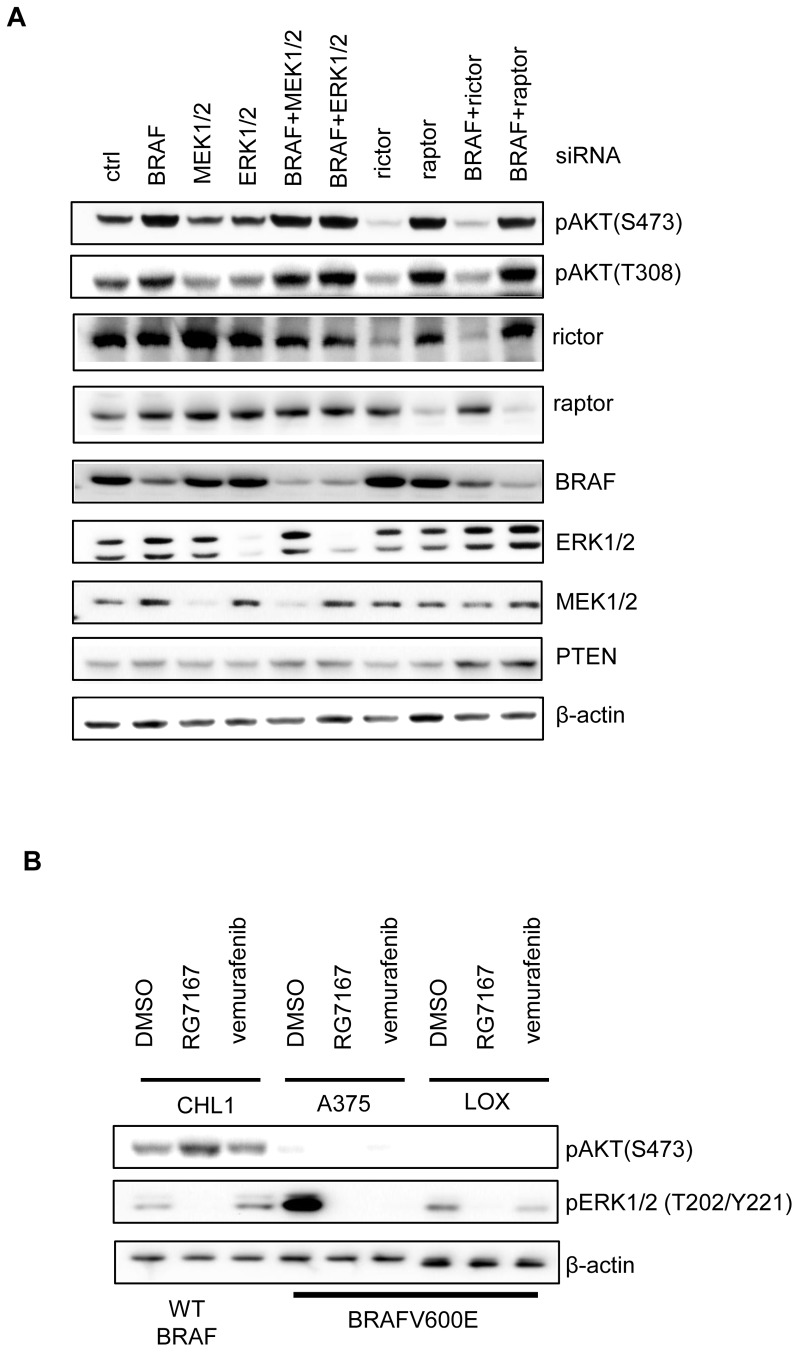
BRAFV600E suppresses AKT phosphorylation through rictor, but independent of MEK, ERK and its kinase activity. (A) Western blot analysis of AKT phosphorylation and PTEN in A375 melanoma cell line 24 hours after knock-down of BRAF, MEK1/2, ERK1/2, rictor, raptor, or combined knock-down. (B) Western blot analysis of AKT and ERK phosphorylation in CHL1, A375 and LOX melanoma cell lines treated with RG7167 or vemurafenib for 4 hours.

As BRAFV600E mutation leads to a constitutive activation of the MAPK pathway, it is possible that suppression of AKT activation in melanoma is mediated through hyperactive ERK signaling. Hyperactive ERK has been shown to phosphorylate TSC2 and impair TSC1/2 functions [24]. As TSC1/2 heterodimers are negative regulators of mTORC1 activities, hyperactive ERK can thus suppress TSC1/2 and activate mTORC1 signaling. Activated mTORC1 signaling will subsequently lead to suppression of pAKT through an internal negative feedback loop that attenuates IGF-1-dependent AKT signaling [18], [25]. To tease apart the molecular mechanism by which BRAFV600E impacts AKT pathway activation, we knocked down individual components of the MAPK pathway and assessed their contribution to AKT signaling in A375 melanoma cells. While knock-down of BRAFV600E readily elevated pAKT, knock-down of MEK1/2 or ERK1/2 showed minimal effects on pAKT (Fig. 4A). Combined knock-down of BRAFV600E and MEK1/2 or BRAFV600E and ERK1/2 showed no difference in the induction of pAKT, compared to that of knock-down of BRAFV600E alone (Fig. 4A). This result suggests that BRAFV600E in melanoma is able to impact AKT pathway independent of downstream MEK and ERK.

As BRAFV600E is a serine/threonine kinase, we then sought to understand if BRAFV600E regulates AKT pathway through its kinase activity. When CHL1 melanoma cells (wild-type BRAF) were treated with BRAF inhibitor vemurafenib, it induced the paradoxical elevation of ERK phosphorylation as expected in wild-type BRAF cells [26]–[28], but triggered no change of pAKT. In A375 and LOX melanoma cells (BRAFV600E), treatment with vemurafenib suppressed ERK phosphorylation, but there was no induction of pAKT (Fig. 4B). This result demonstrated that BRAFV600E impacted AKT pathway independent of its kinase activity, suggesting a structural role of BRAFV600E in the regulation of AKT pathway activation.

### BRAFV600E regulates AKT pathway through rictor (mTORC2) and impairs mTORC2 enzymatic activity

AKT phosphorylation is regulated by PDK1 and mTORC2. PDK1 phosphorylates AKT at Thr308 and partially activates AKT [29]. mTORC2 phosphorylates AKT at Ser473 and stabilizes that of Thr308 [30]. As our results have shown that BRAFV600E suppressed AKT phosphorylation at both Ser473 and Thr308, we sought to explore the possibility that BRAFV600E may exert its effect through mTORC2. In A375 cells, knock-down of rictor, a core component of mTORC2, reduced basal level of pAKT whereas knock-down of raptor, a core component of mTORC1, elevated pAKT, consistent with the reported effects of mTORC2 and mTORC1 on AKT phosphorylation (Fig. 4A). Combined knock-down of BRAF and rictor did not result in elevation of AKT phosphorylation, suggesting that the BRAFV600E effect on AKT pathway is rictor dependent. Interestingly, combined knock-down of BRAF and raptor showed no further elevation of AKT phosphorylation, suggesting that pAKT elevation induced by knock-down of BRAFV600E or raptor may be due to a common regulatory mechanism.

**Figure 5:**
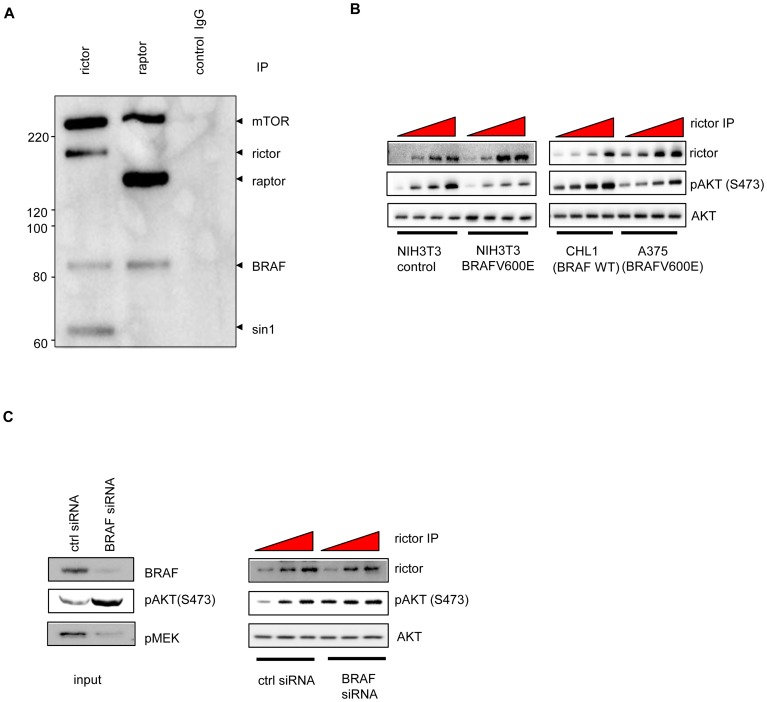
BRAFV600E interacts with rictor complex (mTORC2) and impairs the enzymatic activity. (A) Western blot analysis of BRAF and rictor or raptor complex in respective rictor and raptor immunoprecipitation in A375. (B) Western blot analysis of *in vitro* phosphorylation of recombinant AKT by rictor complex purified from NIH3T3 isogenic pair or CHL1 and A375 melanoma cell lines. (C) Left panel: Western blot analysis of AKT phosphorylation in A375 melanoma cell line 24 hours after control or BRAF siRNA treatment. Right panel: Western blot analysis of *in vitro* phosphorylation of recombinant AKT by rictor complex purified from A375 melanoma cell line 24 hours after control or BRAF siRNA treatment.

To understand how BRAFV600E affects pAKT through mTORC2, we examined a possible interaction of BRAF and mTORC2. When rictor was immunoprecipitated in A375 melanoma cells, BRAF was detected in the rictor complex, in addition to mTOR and sin1 (core components of mTORC2), but not raptor (a component of mTORC1), suggesting that BRAFV600E may interact with mTORC2 directly (Fig. 5A). Notably, we detected the binding of BRAF to rictor-mTOR complex irrespective of BRAF mutation status (data not shown). We were also able to detect BRAF, but not rictor or sin1, in raptor (mTORC1) immunoprecipitates.

Given that BRAFV600E likely interacted with mTORC2 directly, we then purified mTORC2 from various cell lines with either wild-type or mutant BRAF to assess its enzyme activity in an *in vitro* kinase assay. Using an equal amount of recombinant unphosphorylated AKT as a substrate and adding increasing amounts of rictor immunoprecipitates, we found that mTORC2 purified from NIH3T3 (BRAFV600E) cells had lower enzyme activity compared to that purified from the isogenic paired control NIH3T3 cells (Fig. 5B). In melanoma cells, mTORC2 purified from A375(BRAFV600E) also showed lower enzyme activity compared to that purified from CHL1(wild-type BRAF) (Fig. 5B). Furthermore, knock-down of BRAFV600E in A375 melanoma cells enhanced mTORC2 enzyme activity, compared to the control knock-down (Fig. 5C). Together these results suggest that BRAFV600E negatively regulates AKT activities through directly binding to rictor (mTORC2) complex and impairing mTORC2 enzyme activity.

## Discussion

Therapeutic agents targeting oncogenic signaling molecules can induce mechanisms of drug resistance through activation of complex feedback loops. A thorough understanding of the signaling feedback loops will help enable a rational design of combination therapy. In this study, we found a negative role of BRAFV600E in regulating AKT pathway activity. Using an NIH3T3 stable cell line expressing BRAFV600E and various melanoma cell lines (BRAF WT or V600E), we demonstrated that BRAFV600E not only suppresses MEK inhibitor or rapamycin-induced AKT pathway activation but also negatively regulates basal AKT pathway signaling. Importantly, we show that this regulation is through a direct interaction between BRAFV600E and rictor (mTORC2) complex and independent of BRAF kinase activity and downstream MEK and ERK signals. However, the PTEN mutation was sufficient to override the suppressive role of BRAFV600E as in a subset of melanoma cell lines with concurrent BRAFV600E and PTEN deletion, a BRAFV600E effect on pAKT could not be demonstrated. Our finding provides a new interaction node between the MAPK and AKT pathways and suggests a potential resistance mechanism that may emerge in MEK inhibitor-treated melanoma patients.

The detailed molecular mechanism of BRAFV600E modulation of mTORC2 remains to be determined. There is evidence that other RAF family members may mediate signaling transduction in a MEK-independent manner. One example is ASK1, which was shown to be suppressed by CRAF independent of MEK-ERK pathway [31]. Plk1 and Aurora-A were also shown to interact with CRAF at mitotic spindle independent of MEK1/2 [32]. It was proposed that CRAF exerts MEK-independent effects on ASK1, Plk1 and Aurora-A through CRAF scaffolding functions. We show that BRAFV600E regulates the AKT pathway independent of BRAF enzyme activity and we are able to co-immunoprecipitate BRAF with rictor and raptor irrespective of BRAF mutation status. These results suggest a scaffolding function of BRAF to mTORC1 and mTORC2. More studies will be needed to understand how the structural change of BRAFV600E leads to the functional consequence of weakened mTORC2 activity as compared to wild-type BRAF. It is possible that BRAFV600E provides an “active conformation” scaffolding function, as opposed to the “inactive conformation” scaffolding provided by wild-type BRAF, which leads to impaired mTORC2 functions.

**Figure 6:**
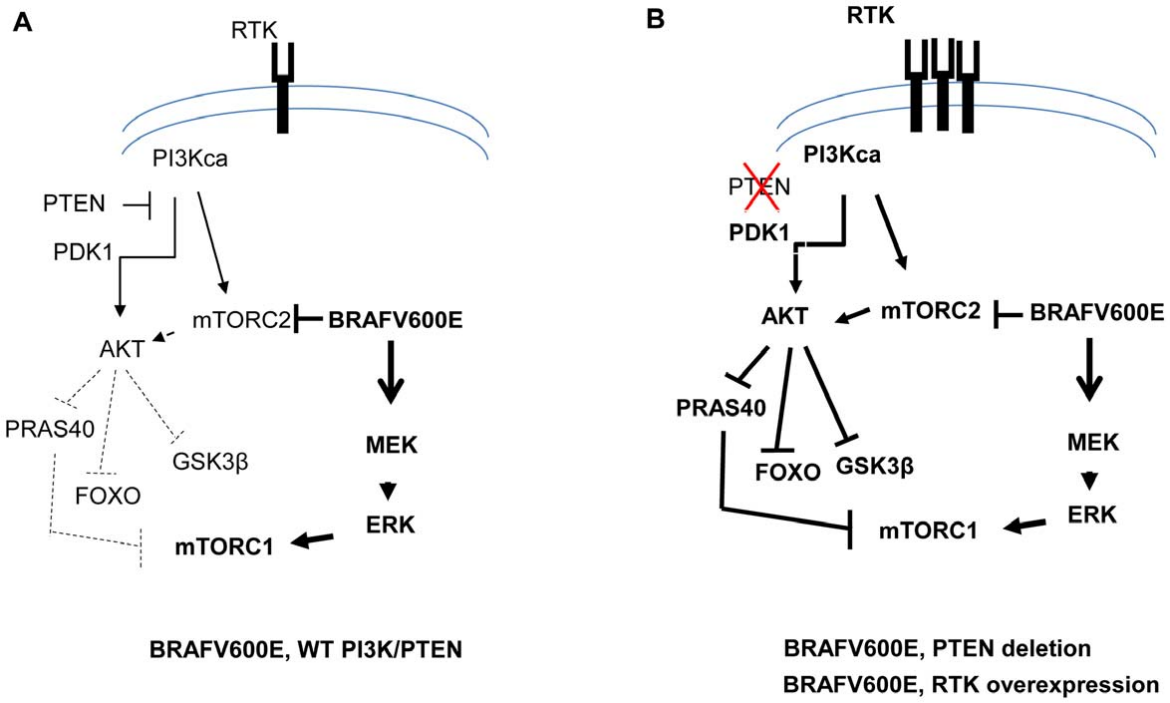
Schematic representations of the cross-talk between MAPK and AKT pathways in cells with different genetic backgrounds. (A) In cells with “wild-type” AKT signaling, the presence of BRAFV600E exerts a negative impact through mTORC2 on AKT phosphorylation and pathway activation. Suppression of AKT activity leads to a de-repression of AKT substrates PRAS40, FOXO and GSK3β due to the reduction of AKT-mediated phosphorylation. (B) In cells where AKT signaling is aberrantly activated (by PTEN loss, PI3K activation, or RTK amplification), the negative impact of BRAFV600E is countered by a dominant input upstream of AKT. Consequently, activated AKT phosphorylates substrates PRAS40, FOXO and GSK3β and suppresses their activity.

Our conclusion that BRAFV600E negatively regulates AKT pathway is consistent with a long-held, but poorly understood, observation in melanoma biology. It has been shown that a high percentage of benign nevi harbor BRAV600E, with no detectable AKT phosphorylation. These nevi likely underwent an initial phase of growth upon acquisition of BRAFV600E but eventually succumbed to senescence. In contrast to the benign nevi, a high percentage of primary melanomas have concurrent mutations in BRAF and components of AKT pathway [2], [33]. Transient transfection of BRAFV600E into primary melanocyte was shown to induce senescence in melanocytes whereas BRAFV600E combined with PTEN silencing elicited melanoma development [34]–[36]. These results, together with our findings, suggest the following scenario in melanoma-genesis: in the nevi, the BRAFV600E mutation is an early event activating pERK, causing an initial growth spurt while suppressing pAKT, potentially leading to the subsequent senescence. Acquiring the BRAF mutation alone, therefore, is not sufficient to induce melanocyte transformation. BRAFV600E mutant melanocytes are under constant selective pressure to activate AKT pathway. Over-expression of RTKs [37], silencing of PTEN, or mutation of LKB1 [38] are several possible mechanisms that may allow BRAFV600E melanocytes to bypass BRAFV600-mediated suppression of AKT pathway to become full-blown melanoma. Figure 6 illustrates our proposed model of the cross-regulation between BRAFV600E and AKT pathway on top of the current understanding of the two signaling pathways [39]. In short, the AKT pathway activity will depends on both “direct input” from AKT upstream signals and “indirect input” from BRAFV600E. In conditions where “direct input” from AKT upstream signals is low, the phosphorylation of AKT and of AKT substrates could be suppressed by BRAFV600E (Fig. 6A). In contrast, in conditions where AKT upstream signal is aberrantly activated by either PTEN loss, or PI3KCA mutation, or RTKs amplification, a strong activation of upstream signals induces AKT phosphorylation and AKT pathway activation, despite the presence of BRAFV600E (Fig. 6B).

Several previous studies have already suggested that BRAFV600E may negatively regulate AKT pathway. LKB1-AMPK signaling is a negative regulator of AKT pathway and has been shown to be modulated by BRAFV600E in melanomas [40], [41]. Maddodi N et al. has recently shown that BRAFV600E induces autophagy and suggested that it is through suppression of AKT pathway [42]. Our study provides new evidence on how BRAFV600E may negatively influence AKT pathway. It is likely that BRAFV600E could negatively impact AKT pathway through multiple mechanisms.

There is accumulating evidence that MAPK and AKT pathways intersect at multiple levels. RAS activates both pathways through interaction with RAF and PI3K respectively. AKT modulates the MAPK pathway through AKT-dependent phosphorylation of BRAF and CRAF. ERK regulates AKT pathway through restraining either upstream GAP1 or downstream TSC1/2 activities. Our study demonstrates a new regulatory mechanism between MAPK pathway and AKT pathway in that BRAFV600E negatively regulates the AKT pathway through interacting with the rictor (mTORC2) complex. This new regulatory mechanism may provide an explanation of why a subset of melanoma cell lines are exquisitely sensitive to MEK inhibition. Our study adds an additional rationale to support combination therapies with RAF/MEK inhibitors and PI3K/AKT/mTOR inhibitors for the treatment of melanoma patients.

## Materials and Methods

### Cell lines and reagents

All cell lines were purchased from the American Type Culture Collection (ATCC). A375, CHL1, SK-MEL-1, A2058, HT-144, and WM266-4 cell lines were grown in DMEM with 10% FBS. LOX cell line was grown in RPMI with 10% FBS. All cell culture reagents were purchased from Life Technologies. All primary antibodies were purchased from Cell Signaling Technology except the following: anti-BRAF from Santa Cruz Biotechnology, anti-CRAF from BD Biosciences, anti-rictor and anti-raptor from Bethyl Laboratories, anti-sin1 from Millipore and anti-β-actin from Sigma-Aldrich. Secondary antibodies were purchased from Cell Signaling Technology and Thermo Scientific. The MEK inhibitor RG7167 was synthesized at Chugai Pharmaceuticals and the BRAF inhibitor vemurafenib was synthesized at Roche. Rapamycin was purchased from EMD Chemicals. siGENOME siRNAs and the transfection reagents were purchased from Thermo Scientific and transfected according to manufacturer's protocols. Transient transfection of CHL1 was conducted with X-tremeGENE 9 DNA transfection reagent (Roche). Plasmids expressing wild-type BRAF or BRAFV600E were purchased from OriGene Technologies.

### Generation of NIH3T3 stable clones (vector-control and BRAFV600E)

Human BRAFV600E cDNA was cloned into pcDNA3-based vector (Promega) and plasmids were transfected into parental NIH3T3 cells (ATCC) for selection. Transfected cells were grown in DMEM with 10% calf serum and 1 mg/ml Geneticin and single clones were selected. Five representative clones were further expanded in DMEM with 10% calf serum and 0.5 mg/ml Geneticin for subsequent experiments.

### Compound treatment and siRNA knockdown studies

All cells were seeded ∼30% confluency 1 day before compounds or siRNAs were applied. Cells were harvested at designated times for compound treatment or 18–24 hours after siRNA transfection. Most samples were collected under subconfluency (<70%) to minimize cell contact-induced change in AKT phosphorylation.

### Immunoprecipitations and kinase assays

For rictor and raptor complex purification, the immunoprecipitation lysis buffer (IP buffer) was prepared as described previously [30]. Rictor and raptor complexes were purified using antibodies from Bethyl laboratories. For *in vitro* rictor complex kinase assay, rictor complex was purified from cells lysed with IP buffer. The immunoprecipitates were then captured with protein A agarose beads (Millipore) and incubated in rictor-mTOR kinase buffer with inactive AKT1 (Upstate Biotechnology) as described previously [30].

### Western blot analysis

Western blotting was carried out as previously described [16] with the following exceptions: for the analysis of rictor and raptor complexes in immunoprecipitation, complex components were blotted simultaneously in the IP product by mixing together the primary antibodies against each component. Clean-blot IP Detection HRP-conjugated secondary antibody (cat#21230, Thermo Scientific) was used to detect both rabbit and mouse primary antibodies simultaneously.

### *In vitro* growth kinetics and *in vivo* xenograft tumor model

For anchorage independent growth assay, cells were plated in 0.4% agar in DMEM containing 20% FBS and allowed to grow for 2–3 weeks before counting the colony. For low serum/growth factor assay, cells were seeded in the regular medium overnight and subsequently grew in the medium with 0.1% FBS. The CellTiter-Glo Luminescent Cell Viability assay (Promega) was used to assess cell proliferation at designated time points. The *in vivo* xenograft model was conducted as previously described [16].

## Supporting Information

Figure S1 **Western blot analysis of the time course response of AKT, MEK and ERK phosphorylation in NIH3T3 isogenic pair treated with RG7167.**
(TIF)

Figure S2 Top left panel: Western blot analysis of MEK and ERK phosphorylation in NIH3T3 isogenic pair. Top right panel: Growth kinetics analysis of NIH3T3 isogenic pair in low serum growth medium in 2D cell culture plates. Lower left panel: Analysis of the anchorage dependency of NIH3T3 isogenic pair in soft agar assay. Lower right panel: Growth kinetics analysis of NIH3T3 isogenic pair in nude mice xenograft model.
(TIF)

Figure S3 **Western blot analysis of AKT phosphorylation in A2058 and HT144 melanoma cell lines 24 hours after knock-down of BRAF.**
(TIF)

## References

[1] Davies H, Bignell GR, Cox C, Stephens P, Edkins S, et al (2002) Mutations of the BRAF gene in human cancer. Nature 417 6892 949–54. 12068308 10.1038/nature00766

[2] Slipicevic A, Holm R, Nguyen MT, Bohler PJ, Davidson B, et al (2005) Expression of activated AKT and PTEN in malignant melanomas: relationship with clinical outcome. Am J Clin Pathol 124 4 528–36. 16146807 10.1309/YT58WWMTA6YR1PRV

[3] Karbowniczek M, Spittle CS, Morrison T, Wu H, Henske EP (2007) mTOR is activated in the majority of malignant melanomas. J Invest Dermatol 128 4 980–7. 17914450 10.1038/sj.jid.5701074

[4] Chapman PB, Hauschild A, Robert C, Haanen JB, Ascierto P, et al (2011) Improved survival with vemurafenib in melanoma with BRAF V600E mutation. N Engl J Med 364 26 2507–16. 21639808 10.1056/NEJMoa1103782 PMC3549296

[5] Solit D, Garraway LA, Pratilas CA, Sawai A, Getz G, et al (2006) BRAF mutation predicts sensitivity to MEK inhibition. Nature 439 7074 358–62. 16273091 10.1038/nature04304 PMC3306236

[6] Kirkwood JM, Bastholt L, Robert C, Sosman J, Larkin J, et al (2012) Phase II, Open-Label, Randomized Trial of the MEK1/2 Inhibitor Selumetinib as Monotherapy versus Temozolomide in Patients with Advanced Melanoma. Clin Cancer Res 18 2 555–567. 22048237 10.1158/1078-0432.CCR-11-1491 PMC3549298

[7] Infante JR, Falchook GS, Lawrence DP, Weber JS, Kefford RF, et al (2011) ASCO Meeting Abstracts CRA8503.

[8] McCubrey JA, Miella M, Tafuri A, Martelli AM, Lunghi P, et al (2008) Targeting the RAF/MEK/ERK pathway with small molecule inhibitors. Curr Opin Investig Drugs 9 6 614–30. 18516761

[9] Steelman LS, Stadelman KM, Chappell WH, Horn S, Basecke J, et al (2008) AKT as a therapeutic target in cancer. Expert Opin Ther Targets 12 9 1139–65. 18694380 10.1517/14728222.12.9.1139

[10] Nazarian R, Shi H, Wang W, Kong X, Koya RC, et al (2010) Melanoma acquire resistance to BRAF(V600E) inhibition by RTK or NRAS upregulation. Nature 468 7326 973–7. 21107323 10.1038/nature09626 PMC3143360

[11] Johannessen CM, Boehm JS, Kim SY, Thomas SR, Wardwell L, et al (2010) COT drives resistance to RAF inhibition through MAP kinase pathway reactivation. Nature 468 7326 968–72. 21107320 10.1038/nature09627 PMC3058384

[12] Villanueva J, Vultur A, Lee JT, Somasundaram R, Fukunaga-Kalabis M, et al (2010) Acquired resistance to BRAF inhibitors mediated by a RAF kinase switch in melanoma can be overcome by cotargeting MEK and IGF-1R/PI3K. Cancer Cell 18 6 683–95. 21156289 10.1016/j.ccr.2010.11.023 PMC3026446

[13] Emery C, Vijayendran K, Zipser M, Sawyer A, Niu L, et al (2009) MEK1 mutations confer resistance to MEK and BRAF inhibition. Proc Natl Acad Sci USA 106 48 2041–6. 19915144 10.1073/pnas.0905833106 PMC2777185

[14] Corcoran R, Dias-Santagata D, Bergethon K, Iafrate A, Settleman J, et al (2010) BRAF gene amplification can promote acquired resistance to MEK inhibitors in cancer cells harboring the BRAFV600E mutation. Sci Signal 3 149 ra84. 21098728 10.1126/scisignal.2001148 PMC3372405

[15] Little A, Balmanno K, Sale M, Newman S, Dry J, et al (2011) Amplification of the driving oncogene, KRAS or BRAF, underpins acquired resistance to MEK1/2 inhibitors in colorectal cancer cells. Sci Signal 4 166 ra17. 21447798 10.1126/scisignal.2001752

[16] Wang H, Daouti S, Li W, Wen Y, Rizzo C, et al (2011) Identification of MEK1 (F129L) activating mutation as a potential mechanism of acquired resistance to MEK inhibition in human cancers carrying the BRAFV600E mutation. Cancer Res 71 16 5535–45. 21705440 10.1158/0008-5472.CAN-10-4351

[17] Carracedo A, Ma L, Teruya-Feldstein J, Rojo F, Salmena L, et al (2008) Inhibition of mTORC1 leads to MAPK pathway activation through a PI3K-dependent feedback loop in human cancer. J Clin Invest 118 9 3065–74. 18725988 10.1172/JCI34739 PMC2518073

[18] O'Reilly KE, Rojo F, She QB, Solit D, Mills GB, et al (2006) mTOR inhibition induces upstream receptor tyrosine kinase signaling and activates Akt. Cancer Res 66 3 1500–8. 16452206 10.1158/0008-5472.CAN-05-2925 PMC3193604

[19] Buck E, Eyzaguirre A, Rosenfeld-Franklin M, Thomson S, Mulvihill M, et al (2008) Feedback mechanisms promote cooperativity for small molecule inhibitors of epidermal and insulin-like growth factor receptors. Cancer Res 68 20 8322–32. 18922904 10.1158/0008-5472.CAN-07-6720

[20] Mirzoeva OK, Das D, Heiser LM, Bhattacharya S, Siwak D, et al (2009) Basal subtype and MAPK/ERK kinase (MEK)-phosphoinositide 3-kinase feedback signaling determine susceptibility of breast cancer cells to MEK inhibition. Cancer Res 69 2 565–72. 19147570 10.1158/0008-5472.CAN-08-3389 PMC2737189

[21] Eblen ST, Slack-Davis JK, Tarcsafalvi A, Parsons JT, Weber MJ, et al (2004) Mitogen-activated protein kinase feedback phosphorylation regulates MEK1 complex formation and activation during cellular adhesion. Mol Cell Biol 24 6 2308–17. 14993270 10.1128/MCB.24.6.2308-2317.2004 PMC355870

[22] Dougherty MK, Müller J, Ritt DA, Zhou M, Zhou XZ, et al (2005) Regulation of RAF-1 by direct feedback phosphorylation. Mol Cell 17 2 215–24. 15664191 10.1016/j.molcel.2004.11.055

[23] Lee L, Niu H, Rueger R, Igawa Y, Deutsch J, et al (2009) The safety, tolerability, pharmacokinetics, and pharmacodynamics of single oral doses of CH4987655 in healthy volunteers: target suppression using a biomarker. Clin Cancer Res 15 23 7368–74. 19934286 10.1158/1078-0432.CCR-09-1696

[24] Ma L, Chen Z, Erdjument-Bromage H, Tempst P, Pandolfi PP (2005) Phosphorylation and functional inactivation of TSC2 by Erk implications for tuberous sclerosis and cancer pathogenesis. Cell 121 2 179–93. 15851026 10.1016/j.cell.2005.02.031

[25] Zhang HH, Lipovsky AI, Dibble CC, Sahin M, Manning BD (2006) S6K1 regulates GSK3 under conditions of mTOR-dependent feedback inhibition of Akt. Mol Cell 24 2 185–97. 17052453 10.1016/j.molcel.2006.09.019 PMC1880887

[26] Poulikakos PI, Zhang C, Bollag G, Shokat KM, Rosen N (2010) RAF inhibitors transactivate RAF dimers and ERK signaling in cells with wild-type BRAF. Nature 464 7287 427–30. 20179705 10.1038/nature08902 PMC3178447

[27] Hatzivassiliou G, Song K, Yen I, Brandhuber BJ, Anderson DJ, et al (2010) RAF inhibitors prime wild-type RAF to activate the MAPK pathway and enhance growth. Nature 464 7287 431–5. 20130576 10.1038/nature08833

[28] Heidorn SJ, Milagre C, Whittaker S, Nourry A, Niculescu-Duvas I, et al (2010) Kinase-dead BRAF and oncogenic RAS cooperate to drive tumor progression through CRAF. Cell 140 2 209–21. 20141835 10.1016/j.cell.2009.12.040 PMC2872605

[29] Stephens L, Anderson K, Stokoe D, Erdjument-Bromage H, Painter GF, et al (1998) Protein kinase B kinases that mediate phosphatidylinositol 3,4,5-trisphosphate-dependent activation of protein kinase B. Science 279 5351 710–4. 9445477 10.1126/science.279.5351.710

[30] Sarbassov DD, Guertin DA, Ali SM, Sabatini DM (2005) Phosphorylation and regulation of Akt/PKB by the rictor-mTOR complex. Science 307 5712 1098–101. 15718470 10.1126/science.1106148

[31] Chen J, Fujii K, Zhang L, Roberts T, Fu H (2001) Raf-1 promotes cell survival by antagonizing apoptosis signal-regulating kinase 1 through a MEK-ERK independent mechanism. Proc Natl Acad Sci USA 98 14 7783–8. 11427728 10.1073/pnas.141224398 PMC35419

[32] Mielgo A, Seguin L, Huang M, Camargo MF, Anand S, et al (2011) A MEK-independent role for CRAF in mitosis and tumor progression. Nat Med 17 12 1641–5. 22081024 10.1038/nm.2464 PMC3233644

[33] Pollock PM, Harper UL, Hansen KS, Yudt LM, Stark M, et al (2003) High frequency of BRAF mutation in nevi. Nat Genet 33 1 19–20. 12447372 10.1038/ng1054

[34] Dankort D, Curley DP, Cartlidge RA, Nelson B, Karnezis AN, et al (2009) Braf(V600E) cooperates with Pten loss to induce metastatic melanoma. Nature Genetics 41 5 544–52. 19282848 10.1038/ng.356 PMC2705918

[35] Dhomen N, Reis-Filho JS, da Rocha Dias S, Hayward R, Savage K, et al (2009) Oncogenic Braf induces melanocyte senescence and melanoma in mice. Cancer Cell 15 4 294–303. 19345328 10.1016/j.ccr.2009.02.022

[36] Michaloglou C, Vredeveld LC, Soengas MS, Denoyelle C, Kuilman T, et al (2005) BRAFE600-associated senescence-like cell cycle arrest of human naevi. Nature 436 7051 720–4. 16079850 10.1038/nature03890

[37] Easty DJ, Gray SG, O'Byrne KJ, O'Donnell D, Bennett DC (2011) Receptor tyrosine kinases and their activation in melanoma. Pigment Cell Melanoma Res 24 3 446–61. 21320293 10.1111/j.1755-148X.2011.00836.x

[38] Guldgerg P, thor Straten P, Ahrenkiel V, Seremet T, Kirkin AF, et al (1999) Somatic mutation of Peutz-Jeghers syndrome gene, LKB1/STK11, in malignant melanoma. Oncogene 18 9 1777–80. 10208439 10.1038/sj.onc.1202486

[39] Laplante M, Sabatini D (2012) mTOR signaling in growth control and disease. Cell 149 2 273–93. 10.1016/j.cell.2012.03.017 PMC3331679 22500797

[40] Zheng B, Jeong JH, Asara JM, Yuan YY, Granter SR, et al (2009) Oncogenic B-RAF negatively regulates the tumor suppressor LKB1 to promote melanoma cell proliferation. Mol Cell 33 2 237–47. 19187764 10.1016/j.molcel.2008.12.026 PMC2715556

[41] Esteve-Puig R, Canals F, Colomé N, Merlino G, Recio JA (2009) Uncoupling of the LKB1-AMPKalpha energy sensor pathway by growth factors and oncogenic BRAF. PLos One 4 3 e4771. 19274086 10.1371/journal.pone.0004771 PMC2651576

[42] Maddodi N, Huang W, Havighurst T, Kim K, Longley BJ, et al (2010) Induction of autophagy and inhibition of melanoma growth in vitro and in vivo by hyperactivation of oncogenic BRAF. J Invest Dermatol 130 6 1657–67. 20182446 10.1038/jid.2010.26 PMC2869390

