# Supplementary figures and images for: BRAFV600E Negatively Regulates the AKT Pathway in Melanoma Cell Lines
Source: PLoS One. 2012 Aug 3;7(8):e42598. doi: 10.1371/journal.pone.0042598 (PMC3411810; doi:10.1371/journal.pone.0042598)

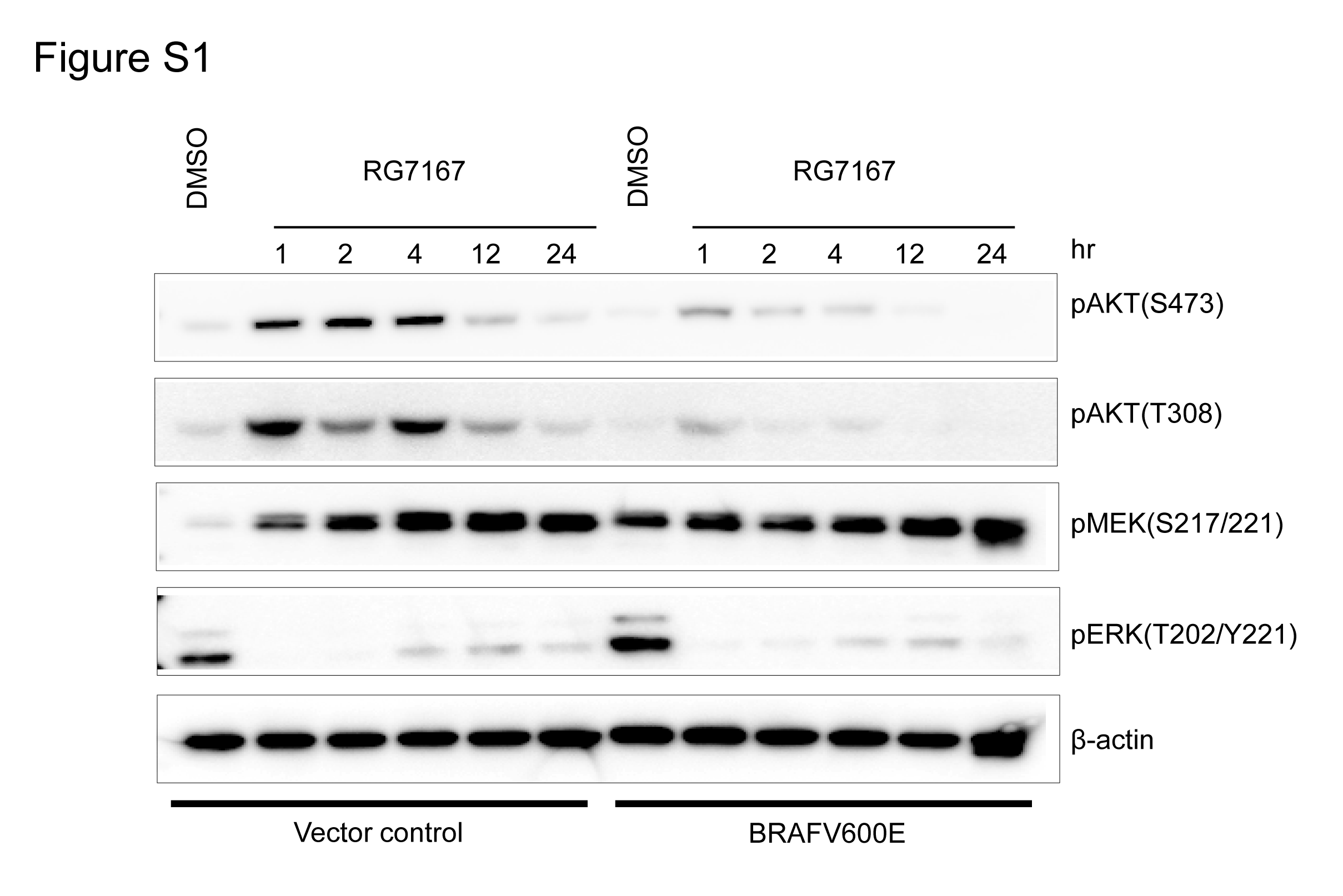

Supplement: Figure S1 — Western blot analysis of the time course response of AKT, MEK and ERK phosphorylation in NIH3T3 isogenic pair treated with RG7167. (TIF) [file pone.0042598.s001.tif]

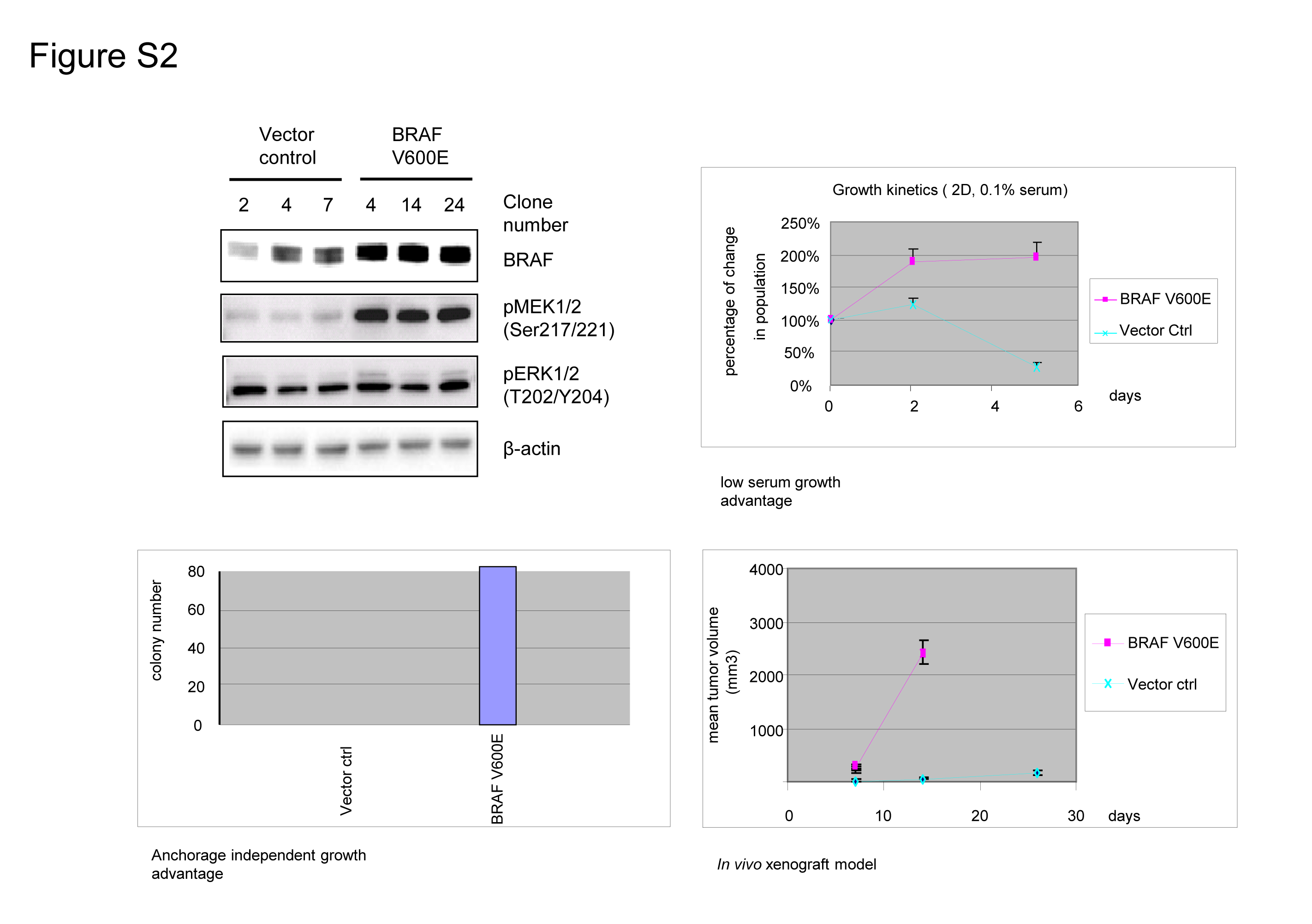

Supplement: Figure S2 — Top left panel: Western blot analysis of MEK and ERK phosphorylation in NIH3T3 isogenic pair. Top right panel: Growth kinetics analysis of NIH3T3 isogenic pair in low serum growth medium in 2D cell culture plates. Lower left panel: Analysis of the anchorage dependency of NIH3T3 isogenic pair in soft agar assay. Lower right panel: Growth kinetics analysis of NIH3T3 isogenic pair in nude mice xenograft model. (TIF) [file pone.0042598.s002.tif]

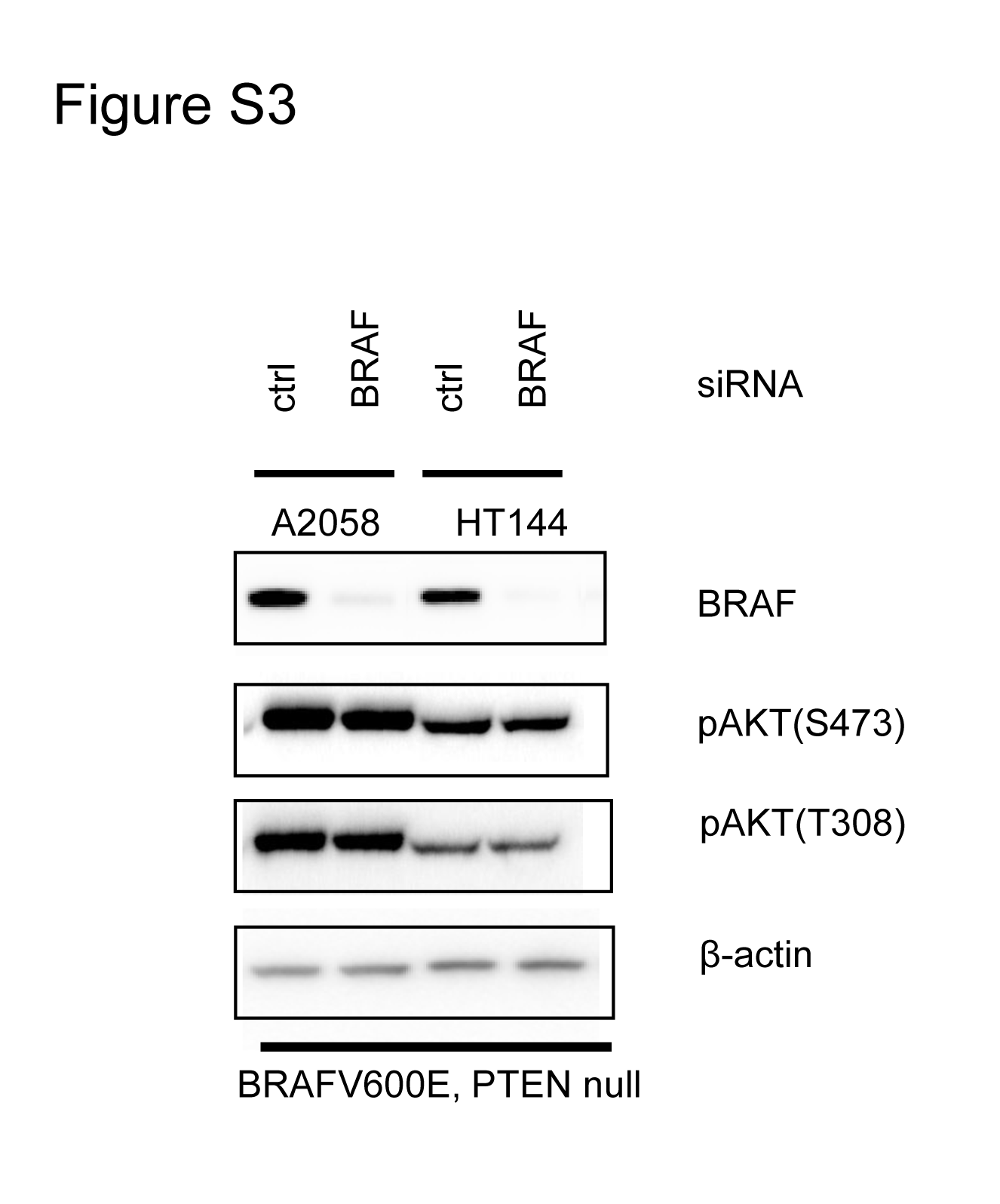

Supplement: Figure S3 — Western blot analysis of AKT phosphorylation in A2058 and HT144 melanoma cell lines 24 hours after knock-down of BRAF. (TIF) [file pone.0042598.s003.tif]
